# Targeting pro-inflammatory cytokines following joint injury: acute intra-articular inhibition of interleukin-1 following knee injury prevents post-traumatic arthritis

**DOI:** 10.1186/ar4591

**Published:** 2014-06-25

**Authors:** Bridgette D Furman, Daniel S Mangiapani, Evan Zeitler, Karsyn N Bailey, Phillip H Horne, Janet L Huebner, Virginia B Kraus, Farshid Guilak, Steven A Olson

**Affiliations:** 1 Department of Orthopaedic Surgery, Duke University Medical Center, Box 3389, Durham, NC 27710, USA; 2 Department of Medicine, Duke University Medical Center, Box 104775, Durham, NC 27710, USA

## Abstract

**Introduction:**

Post-traumatic arthritis (PTA) is a progressive, degenerative response to joint injury, such as articular fracture. The pro-inflammatory cytokines, interleukin 1(IL-1) and tumor necrosis factor alpha (TNF-α), are acutely elevated following joint injury and remain elevated for prolonged periods post-injury. To investigate the role of local and systemic inflammation in the development of post-traumatic arthritis, we targeted both the initial acute local inflammatory response and a prolonged 4 week systemic inflammatory response by inhibiting IL-1 or TNF-α following articular fracture in the mouse knee.

**Methods:**

Anti-cytokine agents, IL-1 receptor antagonist (IL-1Ra) or soluble TNF receptor II (sTNFRII), were administered either locally via an acute intra-articular injection or systemically for a prolonged 4 week period following articular fracture of the knee in C57BL/6 mice. The severity of arthritis was then assessed at 8 weeks post-injury in joint tissues via histology and micro computed tomography, and systemic and local biomarkers were assessed in serum and synovial fluid.

**Results:**

Intra-articular inhibition of IL-1 significantly reduced cartilage degeneration, synovial inflammation, and did not alter bone morphology following articular fracture. However, systemic inhibition of IL-1, and local or systemic inhibition of TNF provided no benefit or conversely led to increased arthritic changes in the joint tissues.

**Conclusion:**

These results show that intra-articular IL-1, rather than TNF-α, plays a critical role in the acute inflammatory phase of joint injury and can be inhibited locally to reduce post-traumatic arthritis following a closed articular fracture. Targeted local inhibition of IL-1 following joint injury may represent a novel treatment option for PTA.

## Introduction

Osteoarthritis (OA) is a debilitating disease characterized by degenerative changes in articular cartilage, bone, and other surrounding tissues. Of the nearly 27 million Americans with symptomatic OA, an estimated 12% have a post-traumatic etiology, making post-traumatic arthritis (PTA) one of the leading causes of joint disability [1,2]. The financial burden of PTA is significant, as it is estimated to cost the US economy over $7 billion annually in work productivity and medical expenses [1]. Additionally, degenerative arthritis following injury is the most common reason for US service members not returning to active duty [3]. PTA can develop after a variety of joint injuries including soft tissue injuries such as ligament and meniscal tears [4-6], articular impact [7,8] or articular fracture [9]. Articular fractures are of particular interest, as they commonly and predictably cause accelerated joint degeneration [10]. The current standard of care for articular fractures is surgical reduction and fixation. Yet, surgical intervention alone does not prevent the development of PTA. Even with optimal treatment, displaced articular fractures of the lower extremity have exhibited a 10 to 20% incidence of clinically significant arthritic degeneration of joint tissues [11].

The pathogenesis of arthritis following joint trauma is not fully understood, and a variety of factors including chondrocyte death, altered joint mechanics, and inflammation have been implicated in the disease. Following joint injury, elevated synovial fluid levels of pro-inflammatory cytokines, interleukin-1 (IL-1) and tumor necrosis factor-alpha (TNF-α), have been reported with the highest levels observed acutely within the first 24 h after injury [12-15]. However, levels remain elevated for weeks to months post-trauma [14,16-19]. Upregulation of IL-1 and TNF-α may play a significant role in the pathogenesis of PTA, similar to their role in chronic OA of joint tissue in patients without antecedent injury [20,21]. Clinically, cartilage-derived biomarkers are significantly increased within the first month following knee injury [13,22,23], which suggests that significant cartilage damage is occurring within weeks of trauma and that early intervention may influence the long-term sequela of joint degeneration [24].

In order to further characterize arthritis development following joint trauma, we developed a murine model of closed articular fracture of the tibial plateau with progressive arthritic changes in the bone, articular cartilage, and other joint tissues [25] at 8 weeks post-injury in C57BL/6 mice. However, the MRL/MpJ strain of mice known as the superhealer strain was protected from PTA and did not develop degenerative joint changes following articular fracture [19], and exhibited lower levels of both local and systemic inflammation in MRL/MpJ mice compared to C57BL/6 mice [26]. This attenuated inflammatory response may help explain how MRL/MpJ mice are protected from the development of PTA after articular fracture [19]. These findings also suggest that the controlled inhibition of the inflammatory response, either systemically or locally, may represent a novel therapeutic approach for PTA after joint injury.

Targeted blocking of specific pro-inflammatory cytokines has been the focus of several therapies for rheumatic diseases such as rheumatoid arthritis. This approach has led to the development of specific inhibitors of IL-1, such as anakinra (Kineret^®^, Biovitrum, Stockholm, Sweden), a recombinant form of human IL-1 receptor antagonist (IL-1Ra). Endogenous and recombinant IL-1Ra act similarly in competitively inhibiting the binding of both IL-1α and IL-1β to their active receptor [27]. Specific inhibitors of TNF-α have also been developed, such as etanercept (Enbrel^®^, Amgen, Thousand Oaks, CA), a human soluble form of TNF-α receptor II (sTNFRII). sTNFRII binds directly to TNF to block its interaction with cell-surface TNF receptors and modulate the biological responses induced or regulated by TNF. Previous research has shown that administration of either IL-1Ra [28] or TNF inhibitors [29-31] reduces inflammation and cartilage destruction in mouse models of collagen-induced arthritis. IL-1Ra was also shown to enhance meniscal repair in an *in vitro* model [32]. However, it is unclear how these inflammatory mediators if administered following joint injury would influence synovial inflammation and cartilage degeneration following intra-articular trauma. Although a role for IL-1 and TNF-α in the pathogenesis of arthritis has been suggested, the role of pro-inflammatory cytokines in the progression of PTA has not been elucidated. Using these cytokine inhibitors after joint injury may represent a novel therapeutic approach for PTA.

We have previously demonstrated that an acute and prolonged increase in the inflammatory response following joint injury has been associated with the development of arthritis in mice. To investigate the role of inflammation in the progression of arthritis following articular fracture, we targeted pro-inflammatory cytokines first, during the initial acute local response to joint injury, and secondly, systemically for a prolonged 4 week period following injury. Following closed intra-articular fracture in the C57BL/6 mouse knee, the anti-inflammatory agents IL-1Ra (anakinra) or sTNFRII (etanercept) were administered, either locally via a single intra-articular injection immediately following injury or systemically for 4 weeks following injury, and the severity of arthritis was assessed.

## Methods

### Closed articular fracture model in the mouse knee

All procedures were approved by the Duke University Institutional Animal Care and Use Committee. Male C57BL/6 mice (n = 62, Charles River, Wilmington, MA) were obtained at 8 weeks of age and housed until 16 weeks of age, at which time active growth has decreased, and peak bone mass is achieved [33,34]. All animals received a moderate closed articular fracture of the tibial plateau as previously described [25]. Animals were anesthetized and placed on a custom cradle. With the left hind limb in a neutral position of 90° of flexion, a 10-N compressive preload was applied to the tibial plateau using a materials testing system (ElectroForce ELF3200; Bose, Framingham, MA, USA) via a custom indenter. The tibia was then loaded in compression at a rate of 20 N/second to induce fracture. The displacement of the indenter was limited to 2.7 mm during loading, which results in moderately severe fractures. No fixation or surgical intervention was employed. Animals were given analgesic (buprenorphine, 48 h) following fracture induction and allowed immediate *ad libitum* weight-bearing and motion. All animals were sacrificed 8 weeks post injury.

### Acute local and prolonged systemic drug delivery

Either saline, IL-1Ra (anakinra, Kineret^®^, Biovitrum, Stockholm, Sweden) or sTNFRII (etanercept, Enbrel^®^, Amgen, Thousand Oaks, CA, USA) were delivered following fracture (n = 6 to 9 per group). For the local inhibition group, animals received a single intra-articular injection immediately following fracture of saline (6 μl, n = 7), IL-1Ra (0.9 mg, n = 8), or sTNFRII (0.3 mg, n = 8). For the systemic inhibition group, saline, IL-1Ra, or sTNFRII was administered for 4 weeks following fracture. Due to the short half-life and method of action of IL-1Ra, either daily subcutaneous injections or continuous infusion was required. For this study, systemic IL-1Ra was delivered by continuous infusion at a dosage of 1.0 mg/day [35,36] using subcutaneously implanted osmotic pumps (model 2004; Alzet; Durect, Cupertino, CA, USA) [37]. Pumps loaded with either IL-1Ra (n = 9) or saline (n = 9) were implanted immediately following fracture for a duration of 4 weeks. For systemic administration of sTNFRII, the method of delivery was intraperitoneal (IP) injections at a dose of 0.2 mg/day [38,39]. Either sTNFRII (n = 9) or saline (n = 9) was administered three times per week for a duration of 4 weeks starting on the day of fracture. When administered systemically at the indicated dosage, both clinically available drugs have been shown to be effective at reducing inflammatory arthritis in murine models [28,40-45].

### Serum drug levels

Serum levels of delivered IL-1Ra and sTNFRII were measured by collecting blood from the maxillary vein in live mice. To ensure the safety of the animals, blood was collected at 2-week intervals from each animal. For mice receiving local intra-articular drug delivery immediately after fracture, early time points following intra-articular injection were chosen for blood collections, day 1, 3, 14 and 15 or 18 post fracture. Due to limited sample remaining following quantification of delivered IL-1Ra, sufficient quantities of serum from the intra-articular saline group for quantification of sTNFRII were only available at the day-14 time point. For mice receiving systemic sTNFRII or saline delivered via IP injections for 4 weeks following fracture, weekly and bi-weekly time points were chosen for blood collections, day 1, 7, 14, 28 and 42 post fracture. For mice receiving systemic IL-1Ra or saline for 4 weeks following fracture delivered via a subcutaneous osmotic pump, due to technical challenges, blood was only sampled on the last day of drug delivery at 4 weeks post fracture. Blood was centrifuged at 10,000 g for 5 minutes, and serum was stored at -80°C until analyzed. Due to limited volumes of blood collected from live mice, samples were pooled by time point to provide sufficient volume necessary for analysis. Serum levels of delivered drugs were measured using human IL-1Ra and sTNFRII commercially available enzyme-linked immunosorbent assays (ELISA kit; IL-1Ra, DRA00B; sTNFRII DRT200; R&D Systems, Minneapolis, MN, USA). To assess the effect of drug delivery, native levels of mouse IL-1Ra or sTNFRII were quantified in serum obtained at time of sacrifice in those animals that received either drug using commercially available ELISA kits (IL-1Ra, MRA00; sTNFRII, MRT20; R&D Systems).

### Sample and tissue collection

All mice were sacrificed at 8 weeks post fracture. Serum was collected via retro-orbital bleed followed by a cardiac stick. Synovial fluid was collected from both knees using a calcium sodium alginate compound [46]. Serum and synovial fluid were stored at -80°C until analyzed. After sacrifice and limb harvest, both hind limbs were placed in 10% neutral-buffered formalin for 72 h.

### Bone morphology

The knee joints of both fractured and contralateral control limbs were scanned by a micro computed tomography (microCT) system (microCT 40, Scanco Medical AG, Bassersdorf, Switzerland) to assess bone morphology. A hydroxyapatite calibration phantom was used to scale values (mg/cm^3^). Morphometric parameters of fully calcified bone were determined using a direct three-dimensional approach [47,48] in the distal femoral condyles, proximal tibial plateau immediately distal to subchondral bone, and metaphyseal region of tibia beginning at fibular attachment [25]. Parameters reported in the femoral condyles were cancellous bone fraction (bone volume/total volume) for the trabecular bone only and bone mineral density (mg/cm^3^). Parameters reported in the tibial plateau were bone volume (mm^3^), bone fraction, and bone mineral density, and in the tibial metaphysis bone volume and bone mineral density.

### Histological assessment of articular cartilage and synovium

All limbs were decalcified (Cal-Ex Decalcification Solution, Fisher Scientific, Pittsburgh, PA) for 72 h, processed, and paraffin-embedded for histology using a commercially available automated tissue processor (ASP300S, Leica Microsystems, Leica Biosystems, Buffalo Grove, IL). Histological sections were taken at 8 μm in the coronal plane of the joint. Sections that captured the tibiofemoral articulation were selected. In each quadrant the lateral tibia (LT), lateral femur (LF), medial tibia (MT), and medial femur (MF) was evaluated separately. The degree of arthritic changes was assessed from Safranin-O- and fast-green-stained sections using a modified Mankin score [25,49,50]. The maximum possible score was 30 for each quadrant. The scores from all quadrants were summed for a total joint score with a possible maximum joint score of 120. For H&E-stained sections, the degree of synovial inflammation was assessed using a standardized synovitis score [15,51], which evaluates the synovial insertion of each quadrant for synovial lining thickness and cellular density in the synovial stroma. The maximum score for each quadrant was 6. A medial and lateral side synovitis score was summed from the associated quadrants with a maximum score of 12 per side of the joint. A total of three graders, blinded to treatment group, scored all specimens. The mean scores of the three graders were used for statistical analysis. The overall inter-grader and intra-grader reliability of the synovitis score was evaluated using Krippendorff’s alpha.

### Serum and synovial fluid biomarkers

Due to the limited volumes of blood and synovial fluid collected, it was not feasible to perform analyses in duplicate, therefore for each of the analytes, a single value was obtained. Osteocalcin, a measure of osteoblast activity and bone formation, was measured in mouse serum, diluted 5-fold as directed, using a sandwich ELISA (Biomedical Technologies, Inc, #BT-470, Stoughton, MA, USA). A competitive ELISA was used to quantify C-terminal telopeptides of type I collagen (CTX-I) in mouse serum (IDS, RatLaps EIA, Scottsdale, AZ, USA), which is a measure of osteoclast activity and bone resorption. For detection of type II collagen degradation products of C-terminal telopeptides of type II collagen (CTX-II), a sandwich ELISA (IDS, Serum Pre-clinical Cartilaps) was used. Serum samples were run undiluted as directed. Two different sandwich ELISAs were employed for the determination of free active (FA) transforming growth factor (TGF)-β_1_ (BioLegend, #437707, San Diego, CA, USA) and total TGF-β_1_ (BioLegend, #436707). Samples were run undiluted for FA TGF-β_1_ and diluted 1,000-fold for total TGF-β_1_ as recommended by the manufacturer. Serum and synovial fluid IL-6 levels were measured using a commercially available ELISA kit specific for mouse IL-6 (R&D Systems, M6000B). Serum samples were run undiluted as recommended and synovial fluid samples were diluted 5-fold. Values obtained for synovial fluid samples were multiplied by 5 to account for the assay dilution as well as by 50 to account for the dilution factor introduced by the collection method employed. A competitive ELISA (MD Bioproducts, #M046012, St Paul, MN, USA) was used to quantify cartilage oligomeric matrix protein (COMP) in synovial fluid samples. Samples were diluted 5-fold and final values were multiplied by 5 to account for this dilution as well as by 50 to account for the dilution factor introduced by the collection method employed.

### Statistical analysis

All statistical analyses were performed using Statistica 7 software (StatSoft, Tulsa). Statistical analysis of arthritis severity from the Mankin score was performed using repeated measure two-way analysis of variance (ANOVA) (with the Fisher least significant difference (LSD) test post hoc) with limb as the repeated factor and treatment group as the second factor. Statistical analysis of synovitis, serum, and synovial fluid measures were performed by non-parametric analyses using Wilcoxon matched pairs for comparison of fractured and contralateral control limbs, and Kruskal-Wallis ANOVA for comparison among treatment groups. Statistical analysis of changes in bone morphology were performed using the one-sample *t*-test to test if differences between fractured and contralateral control limbs were significantly different to 0, and one-way ANOVA (with Fisher LSD test post hoc) to test for significant differences among treatment groups. Spearman’s rank-order correlation coefficient, *r*_s_, was determined to assess the strength of the association between outcome measures. For all tests, significance was reported at the 95% confidence level.

## Results

### Treatment groups following closed articular knee fracture in mice

Moderate articular fractures that were typically located in the lateral aspect of the tibial plateau were successfully created in 60 mice. However, not all mice completed the study at 8 weeks post fracture for analysis due to various complications including pump implantation and health issues unrelated to treatment. Therefore, at 8 weeks post fracture (the time of sacrifice), we had a sample population of n = 52 consisting of the following: intra-articular local saline (n = 7), intra-articular IL-1Ra (n = 8), intra-articular sTNFRII (n = 8), systemic saline via osmotic pump (n = 7), systemic IL-1Ra via osmotic pump (n = 6), systemic saline via IP injections three times weekly (n = 9) and systemic sTNFRII via IP injections three times weekly (n = 7). For all outcome measures, the two methods of systemic administration of saline were compared. Because no significant differences were detected for any of the outcome measures in mice receiving systemic saline via osmotic pump or IP injections, all mice receiving systemic administration of saline were combined for statistical analyses.

### Longitudinal serum quantification confirms drug delivery during treatment

For mice receiving local intra-articular drug delivery, serum levels were quantified at early time points (Table 1). Levels of both delivered IL-1Ra and sTNFRII peaked on day 1 and remained detectable at day 3. However, both were undetectable in the circulation by day 14. Serum quantification of locally delivered drugs demonstrated that local intra-articular injection of either IL-1Ra or sTNFRII into the joint space subsequently migrated to the serum on days 1 and 3 and was cleared fully from the circulation by day 14.

**Table 1:** Serum drug levels during treatment

	**Time, days**	**Local saline**	**Local drug**
**Serum levels of locally delivered IL-1Ra, pg/ml**	1	180	1,460
	3	151	271
	14	23	18
	15	6	10
**Serum levels of locally delivered sTNFRII, ng/ml**	1	NA	803
	3	NA	128
	14	0.01	0.2
	18	NA	0.6
**Serum levels of systemically delivered IL-1Ra, pg/ml**	28	3	36,000
**Serum levels of systemically delivered sTNFRII, ng/ml**	1	0.01	5,615
	7	0.01	3,789
	14	0.01	44
	28	0.01	97
	42	0.01	0.11
	56	0.01	0.01

NA- sample was Not Available for testing.

Serum levels of the drugs delivered either locally via a single intra-articular injection immediately following fracture or systemically for 4 weeks following fracture. Delivered drugs were measured in serum using human IL-1Ra and human sTNFRII commercially available ELISAs. Data are presented as mean ± standard deviation.

For mice receiving either systemic saline or IL-1Ra via an osmotic pump, serum levels of delivered IL-1Ra were measured at 4 weeks post fracture, 1 day prior to pump removal (Table 1). Serum levels of delivered IL-1Ra levels were extrapolated to be 32,000 pg/ml, which was above the upper limit of the ELISA range, and the systemic saline group exhibited no detectable levels. For mice receiving either systemic sTNFRII or saline via IP injections three times weekly for 4 weeks post fracture, serum levels of delivered sTNFRII in the systemic sTNFRII group were maximal on day 1 and remained at similar levels on day 7 with lower levels on days 14 and 28 (Table 1). Minimal levels were detected on day 42 and undetectable levels on day 56. The systemic saline group exhibited no detectable levels of sTNFRII. The serum levels confirmed that the systemic sTNFRII group received a substantial systemic dose of the drug. The reduction in day-14 and day-28 serum levels of sTNFRII, despite continued treatment throughout this time course with sTNFRII, was most likely due to the formation of antibodies to the administered sTNFRII. Similar systemic administration of sTNFRII in mice has been effective in reducing inflammatory arthritis, but some mice have developed antibodies to hsTNFRII after 1 week of administration, and all mice developed antibodies after 4 weeks of administration [52]. Levels of mouse IL1-Ra or sTNFRII were quantified in serum at the time of sacrifice to determine if the mice expressed native IL-1Ra or sTNFRII in response to treatment. There was no significant effect of either local or systemic delivery of human IL-1Ra or sTNFRII on native levels at 8 weeks post injury [Additional file 1].

### Reduction in joint degeneration with local IL-1Ra following articular fracture

**Figure 1:**
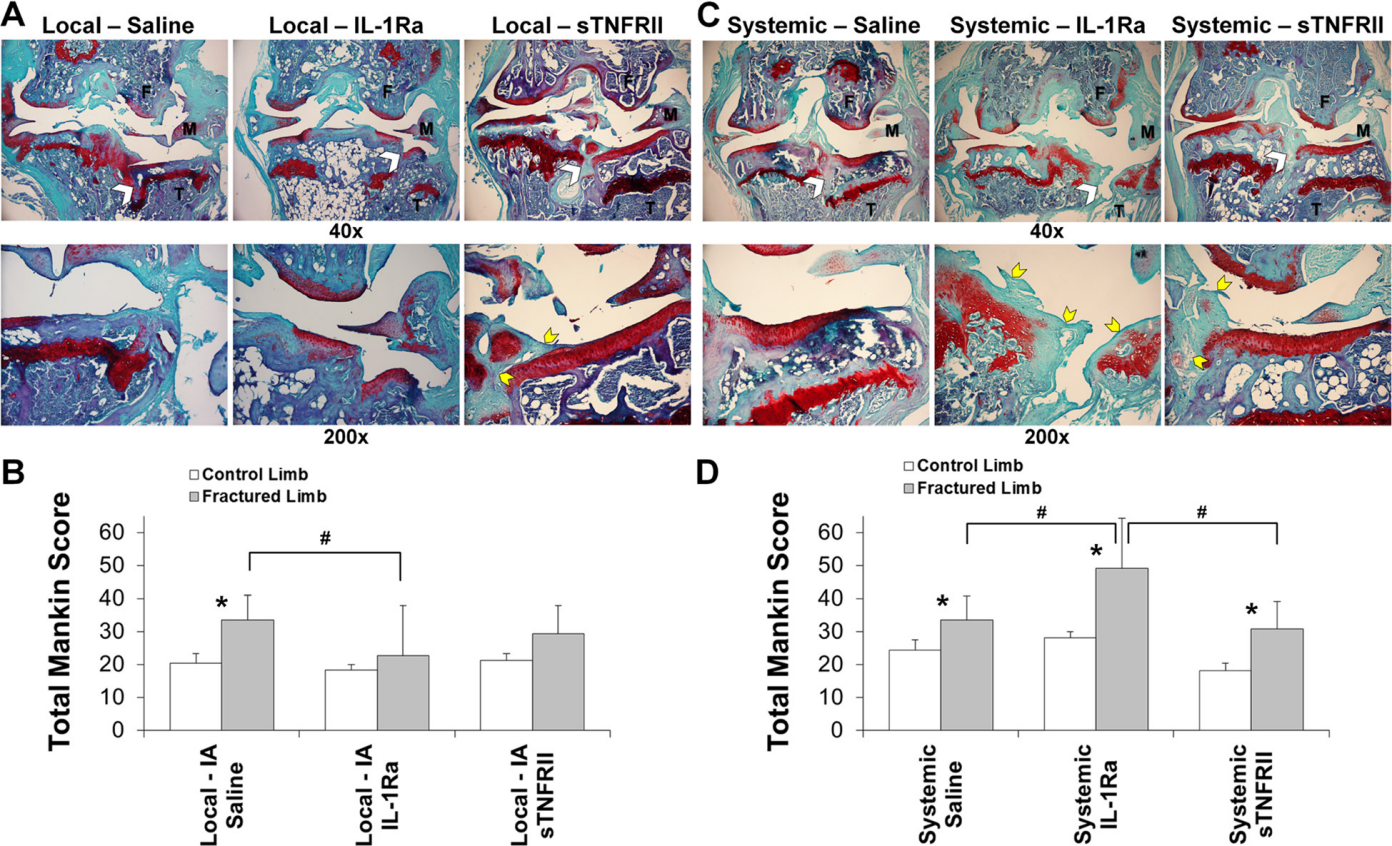
**The effect of local intra-articular and systemic administration of IL-1 receptor antagonist (IL-1Ra) and soluble TNF receptor II (sTNFRII) on degenerative changes in joint tissues following articular fracture.** Histologic images of knee joints stained with Safranin-O (red) and fast green (green) following fracture (F = femur, T = tibia, M = meniscus, white arrow = articular fracture, yellow arrow = fibrocartilage), and total joint Mankin score of arthritic degenerative changes in joint tissues for contralateral control and fractured limbs following local intra-articular (IA) administration of saline, IL-1Ra and sTNFRII **(A-B)** and systemic administration of saline, IL-1Ra and sTNFRII **(C-D)**. Data presented as mean + standard deviation (*significant difference between limbs, ^#^significant differences between treatment groups).

For local intra-articular (IA) delivery following articular fracture, the saline group demonstrated significant degenerative changes in the fractured limb, including loss of cartilage structure and proteoglycan staining on all articular surfaces of the knee joint (Figure 1A). The fractured limb in the local saline group had significantly higher total joint Mankin scores compared to the contralateral control limb (*P* = 0.02) (Figure 1B). For local IL-1Ra, the articular fractures were evident on histologic sections, but there were minimal degenerative changes in the joint with no statistically significant differences in Mankin scores between the fractured and contralateral control limbs (*P* = 0.37). For local sTNFRII, fibrocartilage was frequently found at the fracture site, and some loss of cartilage structure and proteoglycans was observed, but there was no significant increase in Mankin score in the fractured limb compared to the contralateral limb (*P* = 0.18). Local intra-articular IL-Ra resulted in significantly lower Mankin scores in the fractured limb compared to saline (*P* = 0.03) but sTNFRII did not (*P* = 0.38).

Systemic delivery of saline following articular fracture demonstrated similar results to the local intra-articular saline in terms of cartilage degenerative changes. The fractured limb showed loss of cartilage structure and staining with significantly greater Mankin scores compared to the contralateral control limb (*P* = 0.03). Systemic IL-1Ra delivery following articular fracture was associated with significant degenerative changes with frequent complete loss of articular cartilage and the presence of fibrocartilage. In contrast to local treatment with IL-1Ra, systemic treatment with IL-1Ra resulted in significantly higher Mankin scores in the fractured limbs compared to contralateral control limbs (*P* = 0.001). Systemic treatment with sTNFRII was associated with the frequent appearance of fibrocartilage at the fracture site and loss of cartilage structure and proteoglycan staining, with altogether significantly greater Mankin scores compared to contralateral control limbs (*P* = 0.04). Systemic IL-Ra resulted in significantly higher Mankin scores in the fractured limb compared to the fractured limbs in both saline (*P* = 0.0001) and sTNFRII (*P* = 0.007).

### Reduction in synovitis with local IL-1Ra following articular fracture

**Figure 2:**
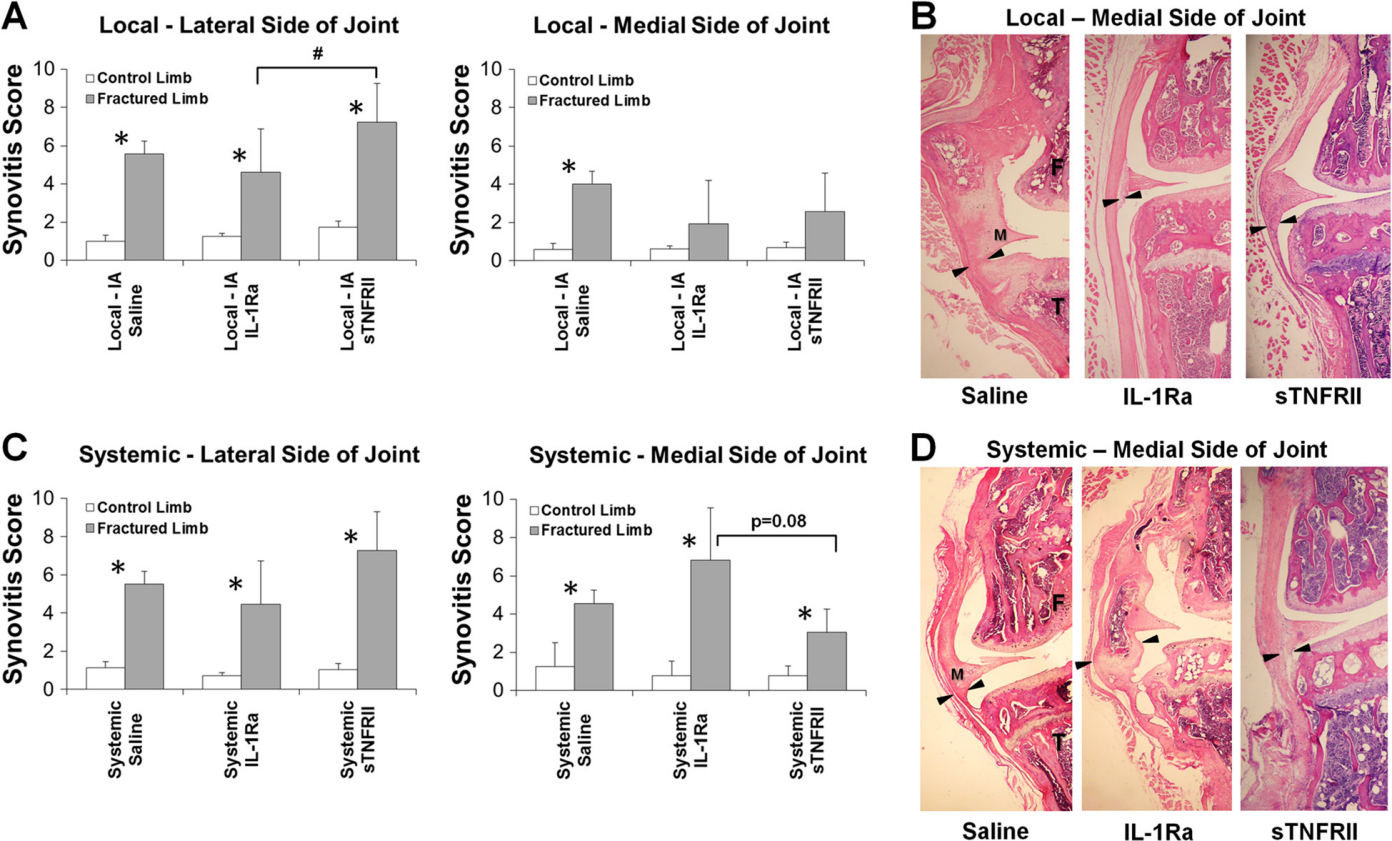
**The effect of local intra-articular and systemic administration of IL-1 receptor antagonist (IL-1Ra) and soluble TNF receptor II (sTNFRII) on synovial inflammation of the knee joint following articular fracture.** Synovitis scores of lateral and medial sides of knee joints for contralateral control and fractured limbs and histologic images of synovium stained with H&E on the medial side of joint with femur (F), tibia (T), and synovial lining near medial meniscus (M) identified by black arrows following local intra-articular (IA) administration of saline, IL-1Ra and sTNFRII **(A-B)** and systemic administration of saline, IL-1Ra and sTNFRII **(C-D)**. Data presented as mean + standard deviation (*significant difference between limbs, ^#^significant difference between treatment groups).

The reliability of the synovitis scores was high for both inter-grader reliability, α = 0.908, and intra-grader reliability, α = 0.958. The articular fractures were typically located in the lateral aspect of the tibial plateau. All fracture groups, independent of treatment, had significantly higher synovitis scores on the lateral side of the fractured limb compared to the contralateral control limb (Figure 2A). This increased synovitis on the lateral side is consistent with proximity to the location of the articular fractures. Local intra-articular IL-Ra resulted in significantly lower lateral synovitis scores in the fractured limb compared to the local sTNFRII fracture limb (*P* = 0.04). However, on the medial side of the joint, the local saline group also demonstrated significantly increased synovitis in the fracture limb compared to the contralateral control limb, indicating global synovial inflammation throughout the joint following fracture. The local IL-1Ra group demonstrated no statistically significant differences in synovitis scores on the medial side of the joint between fractured and contralateral control limbs (Figure 2A). The local IL-1Ra group demonstrated a thin cell-lining layer and low cellular density (Figure 2B). The local sTNFRII group also showed no statistically significant differences in synovitis scores on the medial side of the joint between fractured and contralateral control limbs and demonstrated less synovitis on the medial side of the joint compared to the lateral side of the joint.

Systemic saline demonstrated significant synovitis on both the lateral and medial side of the joint in the fractured limb compared to the contralateral control limb (Figure 2C). However, both systemic IL-1Ra and sTNFRII were different from the local administration in that the fractured limb demonstrated significantly increased synovitis compared to the contralateral control limb on both the lateral and medial side of the joint, which indicated that the global synovial inflammation throughout the joint following fracture was not attenuated with systemic administration of either of these agents. In contrast, systemic IL-1Ra was trending towards increased synovitis on the medial side of the fractured joint compared to systemic sTNFRII (*P* = 0.08).

### Bone morphological changes following fracture with local or systemic administration of saline, IL-1Ra, or sTNFRII

**Figure 3:**
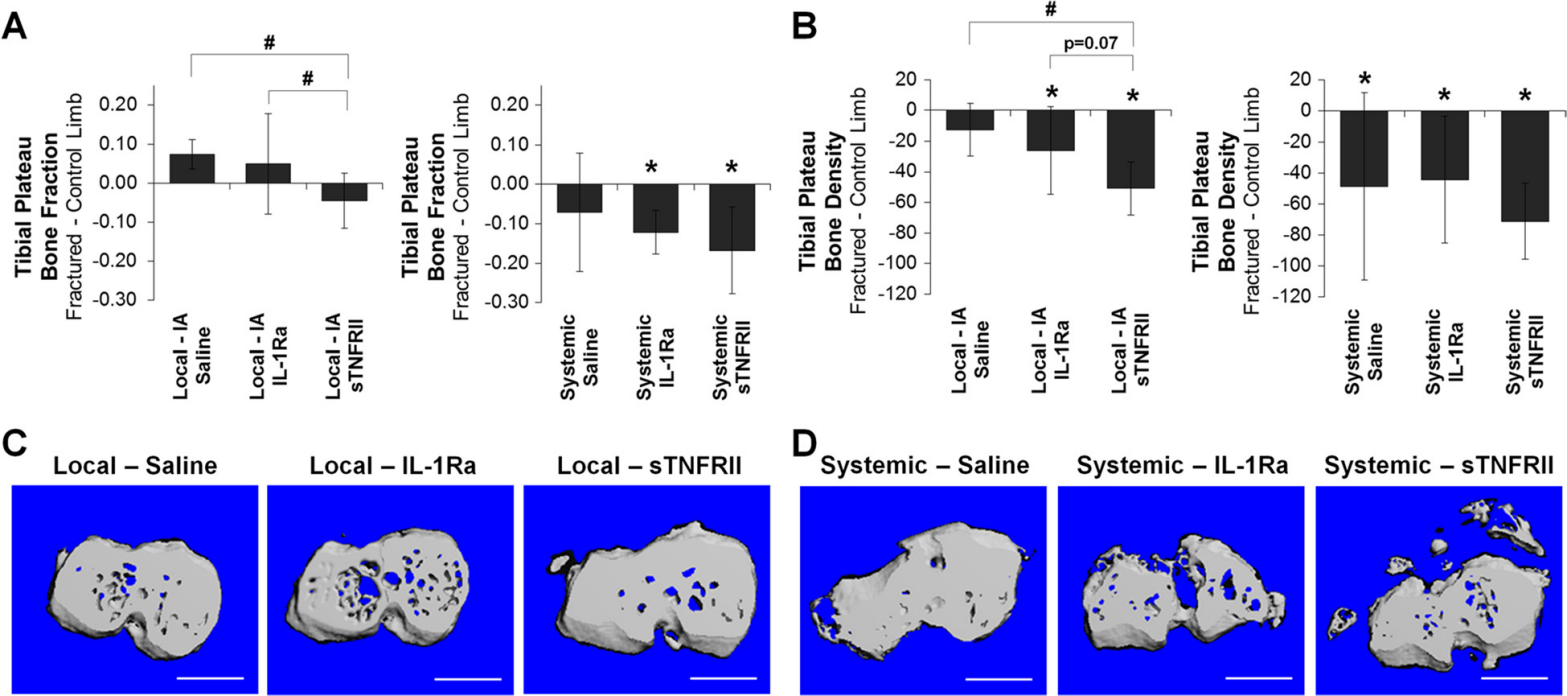
**The effect of local intra-articular and systemic administration of IL-1 receptor antagonist (IL-1Ra) and soluble TNF receptor II (sTNFRII) on bone morphology of the tibial plateau following articular fracture. (A)** Bone fraction and **(B)** bone density of the fractured limb as normalized to the contralateral control limb of each mouse for the tibial. Data presented as mean ± standard deviation (*difference between paired limbs is significantly different to zero, ^#^significant difference between means in treatment groups). Representative axial micro computed tomography (microCT) images of the tibial plateau following **(C)** local intra-articular and **(D)** systemic administration of IL-1Ra and sTNFRII at 8 weeks following articular fracture. Bar, 1 mm.

Articular fracture has been reported to induce decreases in bone volume fraction and bone mineral density in the periarticular tibial plateau [15,19,25]. We assessed the effect of local or systemic administration of saline, IL-1Ra or sTNRII on bone morphology following fracture. Within the tibial plateau, the changes in bone fraction in the fractured limbs normalized to the contralateral control limbs following fracture were not statistically different from zero for local saline, IL-1Ra, or sTNFRII groups (Figure 3A). However, local sTNFRII administration following fracture resulted in significantly reduced tibial plateau bone fraction compared to both local saline and IL-1Ra. Systemic saline demonstrated reduced bone fraction following fracture but the normalized difference between fractured and control limbs was not significantly different than zero. Both systemic IL-1Ra and systemic sTNFRII had reduced tibial plateau bone fraction following fracture that were significantly different than zero, but were not statistically different to systemic saline.

Tibial plateau bone density was reduced following fracture with local and systemic administration of saline, IL-1Ra, or sTNFRII (Figure 3B). No significant reduction in bone density was observed following articular fracture for the local saline group. However, local IL-1Ra and local sTNFRII resulted in a significant reduction of bone density. Changes in bone density were greater with local sTNFRII compared to local saline and trended toward being greater than local IL-1Ra (*P* = 0.07). Groups administered systemic saline, IL-1Ra or sTNFRII had significantly reduced tibial plateau bone density but were not significantly different from each other.

**Figure 4:**
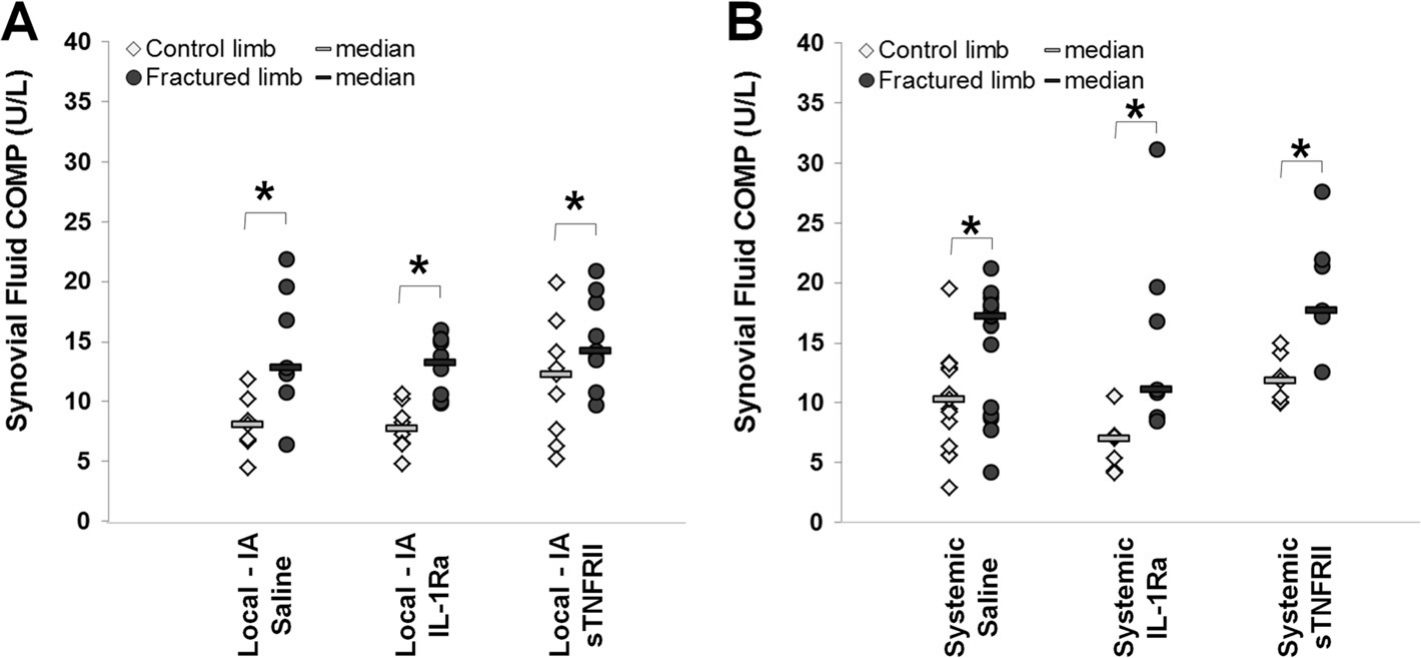
**Synovial fluid levels of Cartilage oligomeric matrix protein (COMP) were elevated with fracture.** Synovial fluid levels following fracture for contralateral control and fractured limbs with **(A)** Local intra-articular (IA) and **(B)** systemic administration of saline, IL-1 receptor antagonist (IL-1Ra) and soluble TNF receptor II (sTNFRII) (*significant difference between limbs).

Within the tibial plateau, local saline and IL-1Ra administered following fracture demonstrated similar bone morphology, whereas local sTNFRII resulted in reduced bone fraction (Figure 3C). Bone morphology changes after fracture were greater with systemic administration of saline, IL-1Ra, or sTNFRII as demonstrated by reduced bone fraction with IL-1Ra and sTNFRII (Figure 4D). Similar bone morphological changes were found in the femoral condyles and the tibial metaphysis [Additional file 2].

### Global joint changes following articular fracture: correlation between histological assessments of arthritic changes, synovial inflammation, and bone morphology following articular fracture

To better characterize PTA disease progression following articular fracture, the relationship between synovial inflammation and arthritic changes in the joint were examined by correlating Mankin and synovitis scores for all groups (Table 2). Interestingly, for the fractured joints, medial Mankin and synovitis scores were significantly correlated (*r*_s_ = 0.61). However, significant correlation was not found in the lateral joint scores for the fractured limbs, nor in the contralateral control joints.

**Table 2:** Global joint changes following articular fracture: correlation between arthritic changes, synovial inflammation and bone morphology

	**Lateral synovitis score**	**Medial synovitis score**			**Lateral synovitis score**	**Medial synovitis score**
**Control limb**			**Fractured limb**			
Mankin total joint score	*r*_s_ = 0.06, *P* = 0.68	*r*_s_ = 0.19, *P* = 0.17	Mankin total joint score		*r*_s_ = 0.10, *P* = 0.47	**r**_**s**_ **= 0.48, *P*** **= 0.001**
Lateral Mankin score	*r*_s_ = 0.16, *P* = 0.26	*r*_s_ = 0.17, *P* = 0.24	Lateral Mankin score		*r*_s_ = 0.06, *P* = 0.68	r_s_ = 0.14, *P* = 0.31
Medial Mankin score	*r*_s_ = -0.07, *P* = 0.60	*r*_s_ = 0.10, *P* = 0.46	Medial Mankin score		*r*_s_ = 0.16, *P* = 0.27	***r***_**s**_ **= 0.61, *P*** **= 0.001**
	**Mankin total joint score**		**Lateral synovitis score**		**Medial synovitis score**	
	**Control limb**	**Fractured limb**	**Control limb**	**Fractured limb**	**Control limb**	**Fractured limb**
Tibial plateau bone volume, mm^3^	***r***_**s**_ **= -0.30, *P*** **= 0.02**	***r***_**s**_ **= -0.39, *P*** **= 0.002**	*r*_s_ = 0.10, *P* = 0.43	*r*_s_ = 0.13, *P* = 0.32	*r*_s_ = 0.11, *P* = 0.38	***r***_**s**_ **= -0.25, *P*** **= 0.05**
Tibial Plateau bone density, mg/cm^3^	*r*_s_ = -0.08, *P* = 0.55	***r***_**s**_ **= -0.29, *P*** **= 0.02**	*r*_s_ = 0.10, *P* = 0.43	***r***_**s**_ **= -0.37, *P*** **= 0.004**	*r*_s_ = 0.10, *P* = 0.46	***r***_**s**_ **= -0.48, *P*** **= 0.001**
Femoral condyle cancellous bone fraction	*r*_s_ = -0.07, *P* = 0.50	***r***_**s**_ **= -0.48, *P*** **= 0.001**	*r*_s_ = 0.09, *P* = 0.36	***r***_**s**_ **= -0.27, *P*** **= 0.006**	*r*_s_ = 0.16, *P* = 0.10	***r***_**s**_ **= -0.45, *P*** **= 0.001**
Tibial metaphysis bone density, mg/cm^3^	*r*_s_ = 0.22, *P* = 0.08	*r*_s_ = -0.11, *P* = 0.40	*r*_s_ = 0.13, *P* = 0.34	***r***_**s**_ **= -0.30, *P*** **= 0.02**	*r*_s_ = 0.06, *P* = 0.60	*r*_s_ = -0.20, *P* = 0.12

Significant Spearman correlations, r_s_, between Mankin scores of arthritic changes, synovitis scores, and bone morphology parameters following articular fracture in the knee indicate a complex interaction among joint tissues with PTA disease progression (correlation coefficients in boldface text are statistically significant, *P* <0.05).

Bone morphological changes following articular fracture also correlated with both severity of arthritic changes and the degree of synovial inflammation (Table 2). Increasing total joint Mankin scores in the fractured limbs correlated with degenerative changes in bone morphology, including decreasing bone volume (*r*_s_ = -0.39) and bone density (*r*_s_ = -0.29) in the tibial plateau and decreasing cancellous bone fraction (*r*_s_ = -0.48) in the femoral condyles. Similarly, synovitis in both the lateral and medial sides of the fractured limbs also inversely correlated with degenerative changes in bone morphology. Although the articular fractures were located on the lateral aspect of the tibial plateau, the medial side demonstrated the greater correlation with periarticular bone in the tibia and femur of the knee joint, including cancellous bone fraction in the femoral condyles (*r*_s_ = -0.48), bone volume (*r*_s_ = -0.25) and bone tissue density (*r*_s_ = -0.48) of the tibial plateau. The association of joint inflammation with arthritic changes and degenerative bone changes on the medial side of the joint, away from the lateral tibial plateau fractures, suggests that global interactions throughout the whole joint may play a role in the progression of arthritis following articular fracture.

### Serum and synovial fluid biomarkers

Biomarkers of bone metabolism showed minimal differences with treatment following fracture. Local intra-articular sTNFRII showed a trend in increased serum osteocalcin (*P* = 0.08) and an increase in serum CTX-I following fracture (Table 3). No differences in either bone marker were found with systemic treatment groups following fracture. Serum levels of FA TGF-β_1_ were not significantly different with local delivery of saline, IL-1Ra, or sTNFRII following fracture. Total TGF-β_1_ was significantly lower with local sTNFRII compared to local saline or local IL-1Ra. However, the ratio of FA/total TGF-β_1_ was not significantly different between local treatment groups. For systemic groups, there was a trend in increasing FA TGF-β_1_ with systemic IL-1Ra delivery (*P* = 0.07), no difference in total TGF-β_1_, and a significantly higher ratio of FA/total TGF-β_1_ with systemic IL-1Ra compared to systemic sTNFRII. Interestingly, TGF-β_1_ levels inversely correlated in both limbs with tibial plateau bone volume (FA TGF-β_1_: control limb, *r*_s_ = -0.33; fractured limb, *r*_s_ = -0.47) and bone fraction (total TGF-β_1_: control limb, *r*_s_ = -0.54; fractured limb, *r*_s_ = -0.51), and the FA/total TGF-β_1_ ratio inversely correlate inversely with tibial plateau bone volume in both limbs (control limb, *r*_s_ = -0.36; fractured limb, *r*_s_ = -0.47).

**Table 3:** Biomarkers and cytokines

	**Local - saline**	**Local - IL-1Ra**	**Local - sTNFRII**	***P *** **(Kruskal-Wallis)**	**Systemic - saline**	**Systemic - IL1-Ra**	**Systemic - sTNFRII**	***P *** **(Kruskal-Wallis)**
Serum osteocalcin, ng/ml	7.3 ± 9.0	11.1 ± 6.7	111.4 ± 199.5^§^	*0.08*	11.1 ± 11.3	10.6 ± 9.9	10.6 ± 9.9	0.93
Serum CTX-I, ng/ml	17.3 ± 7.4^a^	15.9 ± 3.6^a^	34.2 ± 24.6^b^	**0.03**	22.5 ± 16.6	23.0 ± 14.1	19.9 ± 9.9	0.73
Serum free active (FA) TGF-β_1_, pg/ml	19.1 ± 8.2	20.7 ± 10.3	22.4 ± 11.4	0.97	22.8 ± 11.9	34.3 ± 8.0^§^	20.2 ± 19.1	*0.07*
Serum total TGF-β_1_, pg/ml	121,025 ± 45,854^a^	108,251 ± 17,183^a^	84,573 ± 12,760^b^	**0.004**	95,781 ± 24,239	99,868 ± 4,063	90,003 ± 11,717	0.18
Serum FA/total TGF-β_1_ (x 10^-3^)	0.18 ± 0.09	0.19 ± 0.08	0.27 ± 0.14	0.50	0.23 ± 0.10^a,b^	0.34 ± 0.07^a^	0.24 ± 0.25^b^	**0.048**
Serum CTX-II	5.7 ± 10.1	5.7 ± 7.7	3.8 ± 5.3	0.83	2.4 ± 3.1	1.7 ± 4.4	2.7 ± 4.6	0.88
Serum IL-6, pg/ml	32.5 ± 41.5^a,b^	6.3 ± 2.8^a^	67.0 ± 103^b^	**0.004**	17.6 ± 35.6	57.9 ± 78.0^§^	48.3 ± 77.2^§^	*0.09*
SF IL-6 control limb, pg/ml	1,294 ± 319^a,b^	1,348 ± 531^a^	626 ± 505^b^	**0.02**	523 ± 657	1,333 ± 365^§^	1,197 ± 1,675^§^	*0.08*
SF IL-6 fractured limb, pg/ml	1,513 ± 977^a,b^	1,238 ± 299^a^	820 ± 500^b^	**0.04**	730 ± 595^a^	1,370 ± 587^b^	653 ± 486^a^	**0.04**

Serum and synovial fluid (SF) measures of the bone turnover markers osteocalcin and C-terminal telopeptides of type I collagen (CTX-I), cartilage derived CTX-II, joint-tissue-related growth factor TGF-β_1_, and the cytokine IL-6. Data are presented as mean ± standard deviation. Data with different letters are statistically different from each other (*P* <0.05) as measured by Kruskal-Wallis with multiple comparison of the mean ranks. *P***-**values of the measures with significant differences (*P* <0.05) are shown in boldface text. ^*§*^Trend (*P* ≤0.09)*.*

Serum IL-6 levels following fracture were significantly greater with local sTNFRII than local IL-1Ra but not local saline (Table 3). With systemic IL-1Ra and systemic sTNFRII, there was a trend in elevated serum IL-6 levels compared to systemic saline (*P* = 0.09). In contrast to the serum levels, synovial fluid levels of IL-6 were lower in both limbs with local sTNFRII compared to local IL-1Ra but not different to local saline. With systemic administration, the trends in synovial fluid IL-6 levels were similar to serum levels. Synovial fluid IL-6 in the control limbs trended toward being increased with systemic IL-1Ra and sTNFRII compared to systemic saline and was greater in the fractured limb with systemic IL-1Ra compared to both systemic saline and sTNFRII. Synovial fluid levels of IL-6 were not statistically different between control and fractured limbs in all groups.

Synovial fluid levels of COMP were significantly greater in fractured limbs compared to contralateral control limbs in all groups with fracture (Figure 4). However, there were no statistically significant differences in COMP levels with local or systemic administration of saline, IL-1Ra, or sTNFRII following articular fracture for fractured or contralateral control limbs. Synovial fluid COMP levels correlated with increasing synovitis scores in the lateral side of the fractured limbs (*r*_s_ = 0.29) and inversely correlated with cancellous bone fraction in the femoral condyles of the fracture limbs (*r*_s_ = -0.25). Interestingly, COMP synovial fluid levels increased with Mankin scores, but the correlation was not statistically significant (*r*_s_ = 0.177, *P* = 0.18). Serum levels of CTX-II were not significantly different between those receiving local or systemic delivery of saline, IL-1Ra, or sTNFRII following fracture.

## Discussion

Increasing evidence from clinical and animal studies indicates that pro-inflammatory cytokines are elevated following joint injury, yet the specific roles of IL-1 and TNF-α in the development of post-traumatic arthritis are not fully understood. Here, we show that early local inhibition of IL-1 with a single intra-articular injection of IL-1Ra significantly reduced arthritic changes in cartilage, reduced synovitis, and did not alter bone morphology or bone healing after a closed joint fracture in mice. Local inhibition of TNF-α with a single intra-articular injection of sTNFRII had a moderate effect in reducing arthritic changes in the cartilage and synovitis, although not as effectively as IL-1Ra. However, degenerative changes in bone morphology were not reduced with sTNFRII. Systemic infusion of IL-1Ra for 4 weeks post injury, and both local and systemic inhibition of TNF-α with either a single intra-articular injection of sTNFRII or three times weekly injections of sTNFRII for 4 weeks did not reduce arthritic changes, and instead led to significant degenerative changes in bone morphology and evidence of fibrous fracture healing. These results show that intra-articular IL-1, rather than TNF-α, plays a critical role in the acute inflammatory phase following joint injury and can be inhibited locally to reduce post-traumatic arthritis following a closed articular fracture.

While the role of anti-cytokine therapy in PTA remains to be firmly established, its role in autoimmune mouse models of arthritis is relatively well-characterized. For example, continuous high doses of systemic IL-1Ra prevents collagen-induced arthritis (CIA) in DBA/1 mice [35]. Furthermore, chronic overexpression of TNF using a human TNF-transgenic mouse model of TNF-induced arthritis has been shown to be best reduced with a treatment of anti-TNF-antibody (infliximab) and recombinant human IL-1Ra (anakinra) [42]. When administered concurrently, combination therapy of infliximab and anakinra has been shown to block proteoglycan loss in a synergistic fashion [42]. However, systemic cytokine inhibition in the present study did not demonstrate any benefit in reducing PTA, suggesting that future efforts should target intra-articular methods of therapeutic administration. Given that IL-1Ra (anakinra) is commercially available and Food and Drug Administration (FDA)-approved for the treatment of rheumatoid arthritis, the results of the current investigation have broad therapeutic implication and support the extension of translational studies and potential clinical trials in humans. To date, there has been one pilot study of anakinra for acute joint injury in humans [53]; this trial showed that IL-1Ra, administered intra-articularly within the first month following severe knee injury, reduced knee pain and improved function over a 2-week interval. However, the ability of IL-1 inhibition to reduce the development of PTA was not addressed.

In contrast, for treatment of chronic OA, previous clinical trials have reported that neither systemic nor local inhibition of IL-1 were able to reduce clinical symptoms in patients with symptomatic OA of the knee. Systemic inhibition of IL-1 with human monoclonal antibody to IL-1 receptor 1 administered for 12 weeks was found to be well-tolerated, and showed a trend in pain reduction, but the effect was not significant, and the clinical benefit was small [54]. Local inhibition of IL-1Ra with a single intra-articular injection of human recombinant IL-1Ra (anakinra) was also shown to be well-tolerated but only showed a reduction of pain at 4 days and no improvement in OA symptoms compared to placebo at 4, 8, and 12 weeks [55]. Pharmacokinetic data indicate that the mean terminal half-life of IL-1Ra in serum after intra-articular injection is approximately 4 h and is undetectable at 24 h. With the acute local inhibition of IL-1 in this study, we observed less cartilage degeneration and less synovial inflammation. We were also able to detect IL-1Ra in serum up to three days after intra-articular injection, potentially because of decreased clearance due to joint swelling following injury. Our results support a direct role for IL-1 in the acute inflammatory phase of articular injury that can be inhibited at the injury site by exogenous administration of IL-1Ra to reduce PTA.

The systemic IL-1Ra group had the most severe arthritic changes demonstrated by the highest Mankin and synovitis scores. It is important to consider the role of IL-1 in the healing response to fracture. IL-1β was found to stimulate proliferation and differentiation of pre-osteoblasts *in vitro*, as MC3T3-E1 cells produced more mineralized bone matrix when IL-1β was introduced [56]. Moreover, IL-1β can alter the ratio of cartilage volume to callus volume in mice following a diaphyseal tibial fracture within two weeks after the injury [56]. The dosage and timing of administration of the 4-week continuous systemic infusion of IL-1Ra was selected based on previous data showing its effectiveness in ameliorating arthritis in mouse models of inflammatory arthritis [35-37]. However, in our articular fracture model, this strategy of systemic IL-1Ra delivery appears to have altered the healing response. Given our findings, along with previous evidence suggesting a potential role of IL-1 in fracture repair, it could be speculated that IL-1 may transition from playing a negative role in the acute phase of trauma to a positive role in the healing and bone remodeling phase.

In this study, acute local inhibition of TNF-α or systemic inhibition of TNF-α for 4 weeks post injury did not prevent the progression of PTA. From histological evaluations, fibrous healing could be observed at the site of fracture 8 weeks post-injury with administration of sTNFRII. Likewise, bone morphology assessed with microCT indicated that bone fraction and bone density were significantly reduced with administration of sTNFRII. Our findings suggest that inhibiting TNF-α following articular fracture may inhibit fracture healing and bone remodeling. This is consistent with results in a model of simple closed fracture repair in wild-type and TNF-α receptor-deficient mice wherein the absence of TNF-α signaling led to impaired fracture healing [57]. We also found that detrimental changes in bone morphology were correlated to histologic measures of cartilage degeneration and synovial inflammation. This demonstrates the complex inter-relationship between the various joint tissues in the development of post-traumatic arthritis.

We have previously reported reduced bone fraction and bone mineral density following fracture [15,19]. However, these degenerative bone changes appear to be reduced in the local saline group along with the local IL-1Ra group. The data suggest that intra-articular injections of saline may be altering the intra-articular environment in a manner which is beneficial to the periarticular bone. One hypothesis is that intra-articular injections may be diluting catabolic factors or washing out the joint. However, local saline provided no benefit in reducing cartilage degeneration or synovial inflammation. Normal fracture healing involves the upregulation of many inflammatory cytokines and growth factors, and the temporal profiles of these factors are different during the healing process [58,59]. The cytokines IL-1β and TNF-α have also been shown to stimulate the production of active bone morphogenetic protein 2 (BMP-2) [60], which may be involved in the repair process. Understanding the role of such systemic factors found in the circulating serum following trauma may provide insight into articular fracture healing and the development of PTA. Although we saw differences in bone morphology between treatment groups, systemic measures of bone turnover were not significantly different among treatment groups following fracture. We found that markers of both osteoblast and osteoclast activity increased with increasing bone volume or bone fraction in the tibial plateau and metaphysis at 8 weeks post fracture. This time point would represent the remodeling phase of bone repair and has been characterized by high levels of bone resorption and formation markers [61]. Bone turnover markers vary throughout the fracture healing process, and although biochemical markers of bone-turnover have been helpful in understanding and clinical treatment of metabolic bone disease like osteoporosis, their usefulness in assessing fracture healing has not been established [62]. TGF-β1 may play a role in endochondral ossification [63], and is reported to be activated during osteoclast bone resorption [64,65]. In this study, increasing total or free active TGF-β_1_ was associated with decreased bone volume and bone fraction in the tibial plateau. TGF-β_1_ promotes bone formation through chemotactic attraction of osteoblasts and the enhancement of osteoblast proliferation [66]. However, bone-resorbing osteoclasts may help to release free active TGF-β_1_ via their acidic microenvironment [67,68]. Longitudinal assessment of systemic markers of bone turnover and healing may provide more insight into our understanding of articular fracture healing and lead to new methods of assessing interventions that may prevent or mitigate the development of PTA.

Serum IL-6 concentrations were significantly lower in mice that received local IL-1Ra following articular fracture compared to those that received local sTNFRII, and no differences in synovial fluid concentrations of IL-6 were found between fractured and contralateral limbs among any groups. IL-6 is reported to be elevated in synovial fluid following meniscal and ligamentous tears [12,69]. However, IL-6 serum concentrations significantly decrease during fracture healing [70], which may explain the minimal differences among treatment groups at 8 weeks post fracture. Interestingly, serum and synovial fluid IL-6 concentrations increased with increasing cartilage degenerative changes in the medial tibia following articular fracture. *In vitro* studies have demonstrated that IL-6 influences cartilage catabolism with mechanical trauma resulting in increased proteoglycan loss [71]. IL-6 with the soluble IL-6 receptor triggered osteoclast formation and has also been associated with osteoclast-like cell formation in rheumatoid arthritis patients and may contribute to bone resorption [72,73], which supports our observation of an association between increased synovial fluid IL-6 concentrations and decreasing bone fraction in the medial tibial plateau. Serum IL-6 also correlated with increased synovitis scores following articular fracture. The interaction between inflammatory cytokines like IL-1, TNF-α and IL-6 following joint trauma is not well-understood. However, in this mouse model of joint injury, the data suggest a complex relationship between systemic and local biochemical factors and joint pathology of the cartilage, synovium and adjacent bone.

The progression of PTA following joint injury is not well-characterized, and current clinical measures are unable to predict which patients may develop PTA following injury. Identifying serum or synovial fluid molecular biomarkers of degenerative joint changes following injury would provide insight into the early stages of the disease and be a useful and relatively noninvasive diagnostic tool. In this study, synovial fluid COMP was able to distinguish between fractured and contralateral control limbs at 8 weeks post fracture. COMP is an extracellular matrix protein found predominantly in cartilage, but also in synovium, meniscus, ligaments, tendons, and associated with osteoblasts [74-76]. COMP has been suggested as a candidate biochemical molecular marker of arthritis because of its relative specificity to joint tissues. Interestingly, synovial fluid COMP correlated with synovitis and decreased bone fraction but not cartilage degenerative changes. Although COMP has been used as a marker of cartilage turnover [76], COMP has also been reported to be associated with clinical synovitis in patients with knee OA [77], elevated in injured tendon sheath synovial fluid [78], and expressed by adult osteoblasts and may be indicative of metabolic bone activity [74]. In this study, synovial fluid COMP was not significantly different among treatment groups. However, COMP was only measured at a single time point of 8 weeks post fracture. Longitudinal analysis of COMP may provide more insight in future studies.

## Conclusion

This study indicates that acute treatment of an articular fracture with local IL-1Ra therapy can prevent cartilage degeneration and synovial inflammation in the mouse knee. Our investigation supports a direct role for IL-1 in the acute phase of the inflammatory process that follows articular injury. These results further our understanding of the biological mechanisms governing PTA and provide evidence to support the therapeutic benefit of a novel method of treating acute joint injuries that may be used as adjunctive therapy to surgical stabilization.

## Abbreviations

ANOVA: analysis of variance; BMP-2: Bone morphogenetic protein 2; COMP: Cartilage oligomeric matrix protein; CTX-I: C-terminal telopeptides of type I collagen; CTX-II: C-terminal telopeptides of type II collagen; ECM: extracellular matrix; H&E: hematoxylin and eosin; IL-1: interleukin 1; IL-1Ra: IL-1 receptor antagonist; IP: intraperitoneal; LF: lateral femur; LSD: least significant difference; LT: lateral tibia; MF: medial femur; microCT: micro computed tomography; MMP: matrix metalloproteinase; MT: medial tibia; OA: osteoarthritis; PTA: post-traumatic arthritis; r_s_: Spearman’s rank-order correlation coefficient; sTNFRII: soluble tumor necrosis factor receptor II; TGF-β: transforming growth factor beta; TNF-α: tumor necrosis factor alpha.

## Competing interests

Farshid Guilak is an employee of Cytex Therapeutics Inc, and Steven Olson receives research support from Synthes.

## Authors’ contributions

Authors BDF, FG, and SAO contributed to the design of the study. BDF, DSM, and EZ performed all animal experiments. BDF, DSM, EZ, JLH, PHH, and KNB collected all samples, performed analyses, and analyzed data. BDF and DSM drafted the manuscript. EZ, KNB, and PHH helped revising the manuscript. BDF, DSM, JLH, VBK, FG and SAO helped with interpretation of data, statistical analysis, and drafting the manuscript. All authors have given their final approval of the version to be published.

## Supplementary Material

Additional file 1 **Native serum levels of mouse IL-1Ra or soluble TNF receptor II (sTNFRII).** Native levels of mouse IL-1Ra or sTNFRII were quantified in serum obtained at time of sacrifice in those animals that received either local or systemic administration of saline, IL-1Ra or sTNFRII following articular fracture. Click here for file

Additional file 2 **Bone morphology measured by micro computed tomography (microCT).** Bone morphology was assessed for the contralateral control (R) and fractured limbs (L) in the tibial plateau, tibial metaphysis, and femoral condyles. Click here for file
