# Supplementary material for: Targeting pro-inflammatory cytokines following joint injury: acute intra-articular inhibition of interleukin-1 following knee injury prevents post-traumatic arthritis
Source: Arthritis Res Ther. 2014 Jun 25;16(3):R134. doi: 10.1186/ar4591 (PMC4229982; doi:10.1186/ar4591)
Supplement: Additional file 1 — Native serum levels of mouse IL-1Ra or soluble TNF receptor II (sTNFRII). Native levels of mouse IL-1Ra or sTNFRII were quantified in serum obtained at time of sacrifice in those animals that received either local or systemic administration of saline, IL-1Ra or sTNFRII following articular fracture. [file ar4591-S1.pdf]

Additional File 1. Native serum levels of mouse IL-1Ra or sTNFRII. Concentrations of mouse IL-1Ra or sTNFRII were quantified in serum obtained at time of sacrifice in those animals that received either local or systemic administration of saline, IL-1Ra or sTNFRII following articular fracture. Data presented as mean ± standard deviation.

Native mouse IL-1Ra

Group	Serum conc. (pg/ml)
Local-Saline	892±277
Local-IL-1Ra	1132±236
Systemic-Saline	1285±543
Systemic-IL-1Ra	2033±849

Native mouse sTNFRII

Group	Serum conc. (pg/ml)
Local-Saline	5593±2739
Local-sTNFRII	5603±1276
Systemic-Saline	4680±1008
Systemic-sTNFRII	4979±2260
