# Supplementary material for: Targeting pro-inflammatory cytokines following joint injury: acute intra-articular inhibition of interleukin-1 following knee injury prevents post-traumatic arthritis
Source: Arthritis Res Ther. 2014 Jun 25;16(3):R134. doi: 10.1186/ar4591 (PMC4229982; doi:10.1186/ar4591)
Supplement: Additional file 2 — Bone morphology measured by micro computed tomography (microCT). Bone morphology was assessed for the contralateral control (R) and fractured limbs (L) in the tibial plateau, tibial metaphysis, and femoral condyles. [file ar4591-S2.pdf]

Additional file 2. Bone morphology as measured by micro-Computed Tomography (microCT). Bone morphology was assessed for the contra-lateral control (R) and fractured limbs (L) in the tibial plateau, tibial metaphysis, and femoral condyles. Data presented as mean ± standard deviation with statistical analysis of the difference between Fractured (L) – Control (R) limbs; *difference between paired limbs is significantly different than zero (one sample t-test, p<0.05); #significant difference among treatment groups (one-way ANOVA with Fisher LSD post-hoc, p<0.05).

Tibial Plateau – Epiphysis

Bone Volume (BV) (mm^3)

Group	Control (R) limb	Fractured (L) limb	Fractured(L) – Control(R) **significantly different than zero* *#significant difference with treatment*
Local-Saline	0.675±0.056	0.722±0.100	0.047±0.059
Local-IL-1Ra	0.668±0.079	0.667±0.050	0.000±0.061
Local-sTNFRII	0.623±0.035	0.726±0.106	0.103±0.115*
Systemic-Saline	0.583±0.035	0.568±0.066	-0.015±0.151
Systemic-IL-1Ra	0.519±0.038	0.423±0.137	-0.097±0.117
Systemic-sTNFRII	0.603±0.065	0.661±0.174	0.058±0.169

Bone Fraction [Bone Volume (BV)/Total Volume (TV)]

Group	Control (R) limb	Fractured (L) limb	Fractured(L) – Control(R) **significantly different than zero* *#significant difference with treatment*
Local-Saline	0.308±0.043	0.382±0.106	0.074±0.129
Local-IL-1Ra	0.308±0.050	0.357±0.084	0.049±0.071
Local-sTNFRII	0.729±0.032	0.684±0.084	-0.045±0.088 #
Systemic-Saline	0.608±0.111	0.537±0.181	-0.071±0.150
Systemic-IL-1Ra	0.479±0.037	0.357±0.059	-0.122±0.056*
Systemic-sTNFRII	0.670±0.078	0.503±0.125	-0.167±0.109*

Bone Density (mg/cm^3)

Group	Control (R) limb	Fractured (L) limb	Fractured(L) – Control(R) **significantly different than zero* *#significant difference with treatment*
Local-Saline	1137±29	1124±30	-12±28 ┐
Local-IL-1Ra	1143±14	1117±25	-26±17* │ #
Local-sTNFRII	1160±13	1110±26	-51±32* ┘
Systemic-Saline	1152±25	1103±63	-49±60*
Systemic-IL-1Ra	1127±19	1083±77	-45±41*
Systemic-sTNFRII	1148±24	1077±33	-71±24*

Tibial Plateau – Metaphysis

Bone Volume (BV) (mm^3)

Group	Control (R) limb	Fractured (L) limb	Fractured(L) – Control(R)<br>**significantly different than zero*<br>*#significant difference with treatment*
Local-Saline	0.846±0.183	0.930±0.205	0.084±0.257
Local-IL-1Ra	0.846±0.208	1.001±0.180	0.155±0.323
Local-sTNFRII	0.730±0.145	1.164±0.184	0.434±0.108*#
Systemic-Saline	0.731±0.226	0.862±0.225	0.131±0.153* ┐#
Systemic-IL-1Ra	0.518±0.054	0.911±0.165	0.393±0.188* ┘
Systemic-sTNFRII	0.822±0.243	1.061±0.297	0.239±0.216*

Bone Density (mg/cm^3)

Group	Control (R) limb	Fractured (L) limb	Fractured(L) – Control(R)<br>**significantly different than zero*<br>*#significant difference with treatment*
Local-Saline	1024±33	1030±33	6±57
Local-IL-1Ra	1040±47	1022±25	-18±42
Local-sTNFRII	1066±21	1016±27	-50±38*
Systemic-Saline	1047±22	1032±26	-15±38
Systemic-IL-1Ra	1067±39	1011±19	-56±48*
Systemic-sTNFRII	1028±29	993±16	-35±29*

Femoral Condyles – Cancellous Bone

Bone Fraction (BV/TV)

Group	Control (R) limb	Fractured (L) limb	Fractured(L) – Control(R)<br>**significantly different than zero*<br>*#significant difference with treatment*
Local-Saline	0.703±0.072	0.633±0.097	-0.070±0.109*
Local-IL-1Ra	0.739±0.063	0.663±0.076	-0.076±0.089*
Local-sTNFRII	0.699±0.050	0.619±0.100	-0.080±0.088*
Systemic-Saline	0.678±0.081	0.584±0.105	-0.093±0.129*
Systemic-IL-1Ra	0.654±0.069	0.521±0.073	-0.133±0.066*
Systemic-sTNFRII	0.659±0.056	0.515±0.102	-0.145±0.133*

Bone Density (mg/cm^3)

Group	Control (R) limb	Fractured (L) limb	Fractured(L) – Control(R)<br>**significantly different than zero*<br>*#significant difference with treatment*
Local-Saline	1077±32	1068±36	-9±52
Local-IL-1Ra	1051±46	1080±30	29±53*#
Local-sTNFRII	1120±19	1109±30	-11±33
Systemic-Saline	1130±35	1113±28	-17±39*
Systemic-IL-1Ra	1130±26	1118±19	-12±27
Systemic-sTNFRII	1133±32	1115±26	-17±41
